# A Cross-Sectional Study of Factors Influencing Sex Preference of a Child Among Married Women in Reproductive Age Group in a Rural Area of Pune, Maharashtra

**DOI:** 10.4103/0970-0218.69286

**Published:** 2010-07

**Authors:** Madhura Ashturkar, Kevin Fernandez, Harshal T Pandve

**Affiliations:** Department of Community Medicine, Smt. Kashibai Navale Medical College, Narhe, Pune - 411 041, India. E-mail: dr_harshalpandve@yahoo.co.in

Sir,

Sex ratio is an important social indicator to measure the extent of prevailing equity between males and females in the society. It is also a sensitive indicator of development. According to 2001 census, India recorded a sex ratio of 933 females per 1000 males,(1)which is lowest among the ten most populous countries of the world. The preference for son and discrimination against the girl child is almost universal in India and manifest it in many ways. In Maharashtra, the sex ratio has declined from 934 females per 1000 males in 1991 to 922 females in 2001 and the sex ratio for the 0-6 years age group, known as child sex ratio, which is a powerful indicator of social health of any society has also shown a sharp decline from 946 in 1991 to 917 in 2001.(1)According to 2001 census, Pune district has a child sex ratio of 906 which was 943 according to 1991 census.

Owing to declining trend in child sex ratio, a community-based, cross- sectional study was carried out in the rural field practice area of one of the medical college in Pune, among married women in reproductive age group of 15-45 years to determine factors influencing sex preference of a child. Total 210 married women in reproductive age group were selected for the study. Widow, separated and divorcee women were excluded from the study. Simple random sampling technique was used for collection of data. The study subjects were interviewed with structured and pre-tested questionnaire. Mean age of study subjects was 26.75 years. 77.14% of the study subjects were Hindus. 17.5% of the study subjects were illiterate while only 3.81% study subjects were studied up to graduation. 37.62% study subjects were educated up to high school. 90.48% study subjects were housewives, while 5.71% were laborers. 2.86% study subjects had own business and only 2 were employed. Socio-economic status according to modified B. G. Prasad classification showed that majority of the study subjects belonged to class III (33.8%) followed by class IV (31.9%). Mean age at marriage was 18.47 years. The most common reason of son preference was support at old age (57.14%) followed by demand for male child by other family members and community (32.88%). Study by Jai Rup Singh and Centre for Research on Environment Health and Population Activities, Katmandu, Nepal also observed similar reasons for preference to male child.(2,3)The person who had male child will go to heaven (30%) after death was one of the beliefs among study subjects, which was also one of the important reason for preference for the male child. The most common reason for preference to girl child was like for girl child (62.38%). There was no association found between literacy status and socio-economic class of the study subjects and preference to male child in the present study. 22 (10.47%) among 210 subjects had gone for sex determination during their pregnancy for male child. Study showed highly significant association between literacy status and sex determination during pregnancy (*χ^2^value=21.55, P *value*<0.001*). 176 (83.80%) among 210 subjects were aware of the pre-conception – pre-natal diagnostic technique (PC-PNDT) act, while 34 (16.19%) were not aware for the same. No association was found regarding the relation between awareness of PC-PNDT act and study subjects underwent for sex determination during pregnancy (*χ^2^= 3.51, P>0.05*). A UN Convention to Review Status of Women” at UN headquarters, New York by Patel Vibhuti also stated that decline in sex ratio is associated with the increase in female literacy rate.(4)One of the important findings of the study was that 103 (49.04%) of the study subjects had given the history of teen-age pregnancy. Recent National Family Health Survey-3 also shown that 30% women underwent teen-age preganacies.(5)The issue of teen-age pregancy needs attention and further research is required to explore this issue.

In conclusion, the present study has shown that there is a preference for male child in the community. To improve the declining child-sex ratio, it is essential to promote a positive image of the girl child and to value and celebrate the girl child’s life in our family and community.
